# Impact of Telecounselling, Home Monitoring and Exercise on Hospital Readmissions and Quality of Life in Chronic Obstructive Pulmonary Disease: A Randomized Controlled Trial

**DOI:** 10.1111/ijn.70021

**Published:** 2025-05-19

**Authors:** Nejla Köksal, Hanife Durgun

**Affiliations:** ^1^ Dr. Ali Menekse Chest Diseases Hospital Giresun Türkiye; ^2^ Department of Nursing, Faculty of Health Sciences Ordu University Ordu Türkiye

**Keywords:** COPD, hospital readmissions, nursing, quality of life, teleconsultancy

## Abstract

**Background:**

Individuals with Chronic Obstructive Pulmonary Disease (COPD) should engage in regular exercise, emphasizing the importance of home‐based practices.

**Aim:**

This study aimed to investigate the effects of home follow‐up, counselling and exercise training through telecounselling on hospital readmissions and the quality of life of individuals with COPD.

**Methods:**

This was a single‐blind, randomized controlled clinical trial. Individuals in the intervention group were given breathing exercise training and following the training, the patients received weekly exercise programmes. It was added as a control tally on the back page of the training booklet, and a phone call was made to the patients once a week for 12 weeks. No intervention was applied to the control group patients during the 12 weeks. The research report was structured according to CONSORT.

**Results:**

There was a statistically significant difference between the pretest and posttest difference averages of the SF 36 Quality of Life Scale and the number of hospital readmissions of individuals with COPD in the intervention and control groups (*p* < 0.05).

**Conclusion:**

It was determined that exercise training and 12‐week teleconsultancy and home‐based monitoring service applied to the intervention group patients increased the quality of life of individuals with COPD and reduced hospital readmissions.

**Summary**

**What Is Already Known About This Topic?**

- COPD is a progressive lung disease that significantly impacts patients' quality of life and leads to frequent hospital readmissions.
- Regular exercise, particularly home‐based pulmonary rehabilitation, is known to improve respiratory function and overall well‐being in individuals with COPD.
- Telehealth interventions, including remote counselling and follow‐up, have gained attention as a potential method for supporting chronic disease management, but evidence of their effectiveness for individuals with COPD remains limited.

**What This Paper Adds?**

- This study provides evidence that a structured 12‐week home‐based exercise programme combined with telecounselling significantly improves the quality of life in individuals with COPD.
- The intervention group showed a statistically significant reduction in hospital readmissions compared to the control group.
- Regular follow‐up calls and exercise monitoring enhanced patient adherence to prescribed exercises, highlighting the effectiveness of telehealth support.

**The Implications of This Paper**

- The findings support the integration of telecounselling and home‐based exercise programmes into routine COPD management to improve patient outcomes.
- Healthcare policymakers may consider implementing remote follow‐up and structured exercise training as cost‐effective strategies to reduce hospital readmissions.
- Future research can explore long‐term effects and optimize telehealth strategies for broader patient populations, including those with other chronic respiratory conditions.

## 1 Introduction

Known as a major public health problem worldwide, COPD affects almost 300 million people and is the cause of death for 3.2 million people every year (GOLD 2021). COPD is a preventable and curable disease. Symptoms experienced in the disease (dyspnoea, cough, sputum, malnutrition, anorexia, nausea vomiting, constipation, etc.) may cause limitations in the daily life activities of individuals and changes in their emotional, cognitive and physical activities (Hanania and O'Donnell 2019). Repeated hospitalizations may occur because of acute attacks that develop due to symptom increases. Acute exacerbations of COPD negatively affect repeated hospitalizations and the level of COPD, and this situation causes long‐term mortality and morbidity, which negatively affects the quality of life of individuals (Alves et al. 2024; Esteban et al. 2022).

The shortness of breath experienced by individuals with COPD causes fear of death in individuals. Individuals restrict their movements and avoid all kinds of activities to avoid short breathing. However, this restriction that patients make in their movements to protect themselves leads to increased muscle atrophy, and the increase in muscle atrophy leads to increased breath shortness, causing this situation to enter a vicious cycle (GOLD 2021; Ji et al. 2022). In studies, it is stated that high dyspnoea experienced by individuals has negative effects on the ability to perform physical performance; this situation prevents individuals from fulfilling their daily life activities and negatively affects their quality of life (Lee et al. 2020; Wang et al. 2022; Wu et al. 2020). In the management of this condition experienced by individuals with COPD, it is suggested that supporting individuals with regular exercises together with pharmacological treatment can be effective in increasing the quality of life (Saza and Çevik 2020; Tonga and Oliver 2023).

It has been proven by many studies that pulmonary rehabilitation (PR) is the most appropriate method to ensure regular exercise practice in individuals with COPD (Hansen et al. 2020; Wouters et al. 2020; Zeng et al. 2018). Pulmonary rehabilitation is a wide‐ranging, patient‐based, individual‐oriented practice that includes exercise training and behavioural differentiations that should be made into lifelong habits that positively affect both physiological and psychological health, especially in individuals with chronic diseases (Wan et al. 2022). When literature is examined, it is suggested that pulmonary rehabilitation reduces symptoms in individuals with COPD, increases exercise tolerance, increases compliance with the disease, reduces acute exacerbations and should be applied by patients in addition to treatment (Sharma et al. 2023; Troosters et al. 2023) It is stated that pulmonary rehabilitation should consist of applications based on home life, and it is stated that home‐based applications are more effective than hospital‐based applications to increase the adaptation of the individual to daily life (Spielmanns et al. 2023).

Home‐based applications include telehealth applications. Telehealth is defined as a remote access communication network between healthy/patient individuals and health professionals for the purpose of continuous improvement of health services in the provision of treatment and care related to the diagnosis of patients by all health professionals (GOLD 2023). Telehealth includes tools such as smart home services, remote activity monitoring, digital assessment tools, smartphones, wearable technology, audio/video devices and even the use of robots. These tools help health professionals to better monitor their patients, provide support for home care, detect potential health problems early and provide counselling and education for individuals (Söylemez et al. 2023).

In the literature, there are many studies on exercise training applied to individuals with COPD. However, there is no study examining the effect of home‐based monitoring, counselling and exercise training applied with telecounselling method on repeated hospitalization and quality of life of individuals with COPD. Therefore, this study was conducted to examine the effect of home‐based follow‐up, counselling and exercise training applied with telecounselling methods on repeated hospitalization and quality of life in individuals with COPD. The research hypotheses are as follows: H_0_1: Home‐based follow‐up, counselling, and exercise training implemented with telecounselling methods are not effective in reducing repeated hospitalizations of individuals with COPD. H_1_1: Home‐based follow‐up, counselling and exercise training implemented with telecounselling methods are effective in reducing repeated hospitalizations of individuals with COPD. H_0_2: Home‐based follow‐up, counselling and exercise training applied with the telecounselling method are not effective in improving the quality of life of individuals with COPD. H_1_2: Home‐based follow‐up, counselling and exercise training implemented with telecounselling method are effective in improving the quality of life of individuals with COPD.

## 2 Methods

### 2.1 Study Design and Setting

The study used a single‐blind randomized controlled trial and was reported according to the 2017 CONSORT guidelines for the assessment of nonpharmacological interventions. The population of the study consisted of patients hospitalized in the chest department of a state hospital in Dr. Ali Menekse Chest Diseases Hospital, Turkiye.

### 2.2 Participants and Sampling

The eligibility criteria for patients included in the study were as follows, who agreed to participate in the research, diagnosed with COPD, can use a smartphone, oriented and cooperative, no psychiatric disorder such as schizophrenia or dementia that impairs verbal communication, and no communication problems. Exclusion criteria from the study are alcohol or drug addiction, physically disabled and illiterate.

A power analysis was performed with G*Power 3.1.9.4 program to determine the sample size. Using the quality‐of‐life scores in Benton and Wagner's (2013) study evaluating the quality of life of patients with COPD, which was performed according to the two‐way independent samples *t* test in the G*Power 3.1.9.4 program, the effect size was calculated as 0. 5656, Type I error 0.05, Type II error 0.20 (80% power) based on 40 for each group and 80 patients in total, and the study was completed with 80 patients.

### 2.3 Randomization and Prevention of Bias

**FIGURE 1:**
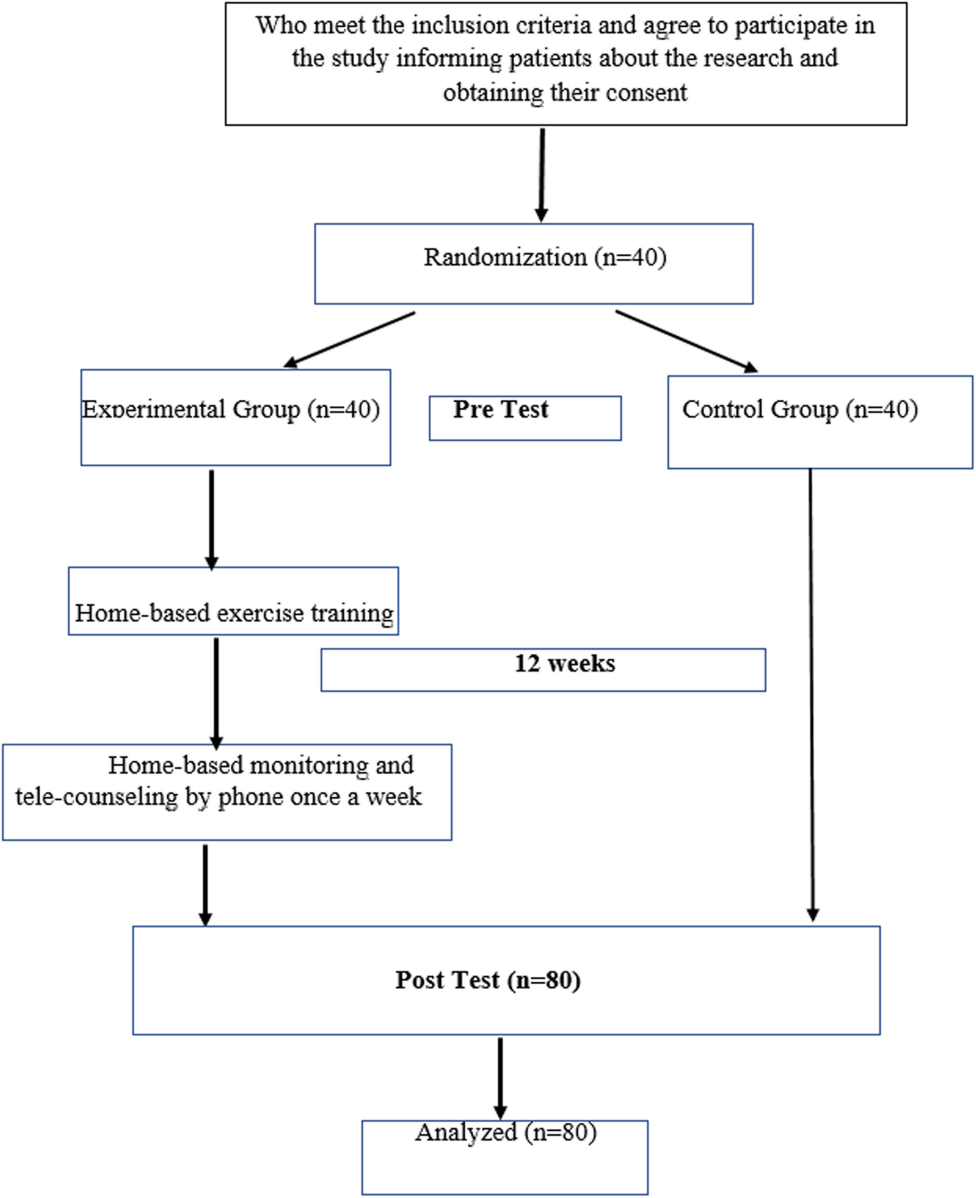
Study flow diagram.

After obtaining consent from patients who met the research criteria and volunteered to participate in the study, pretests were administered. The block randomization method randomly assigned patients to the experimental and control groups (Figure 1). For block randomization, combinations including A and B were created, and six different results were obtained: ABAB (1); ABBA (2); BBAA (3); AABB (4); BAAB (5); and BABA (6) (six combinations). The numbers from 1 to 6 were randomized 20 times in randomizer.org, 80/4 = 20. Combinations were created according to the order generated. The combinations are as follows: BAAB(5); BABA(6); AABB(4); BAAB(5); ABAB(1); ABBA(2); AABB(4); BABA(6); ABBA(2); ABAB(1); BAAB(5); AABB(4); BBAA(3); ABBA(2); BAAB(5); BBAA(3); ABBA(2); BBAA(3); ABAB(1); and AABB(4). In the generated combination, A was assigned as the experimental group and B as the control group by lot method.

The research protocol was based on SPIRIT (Akın and Koçoğlu‐Tanyer 2021), and the reporting of the study was structured according to CONSORT (Tunn et al. 2024). Patients were randomly assigned to the experimental and control groups by block randomization method and did not know that they were in the experimental and control groups. This assignment was done by the researcher who was not the implementer. Group assignments were placed in opaque envelopes and opened by the implementing researcher in turn during randomization. To prevent bias reporting, the data were evaluated by an expert statistician.

### 2.4 Instruments

In the study, data were collected using the Patient Information Form, SF‐36 Quality of Life Scale (Short Form‐SF‐36) and Individuals with COPD Follow‐up Checklist Form. The data were collected immediately after randomization (pretest) and at the end of week 12 (posttest).

The Patient Information Form prepared by the researchers in line with the literature consists of two categories. The first category includes questions about sociodemographic characteristics, and the second category includes questions about the individual's disease and the number of times they had applied to the hospital or emergency room in the last year (Esteban et al. 2022).

SF‐36 Quality of Life Scale (Short Form‐SF‐36) was developed by Ware and Sherbourne (1992) to assess the quality of life of individuals. The scale consists of 36 questions and eight subdimensions including physical functioning, physical role difficulties, emotional role difficulties, energy/vitality, mental health, social functioning, pain and general health perception. Although a total score cannot be obtained from the total scale, each subdimension is scored separately between 0 and 100 and evaluates the last 4‐week status of the individual. A high score on each subscale indicates a high quality of life on that subscale, whereas a low score indicates a poor quality of life in that subscale (GOLD 2021; Ware and Sherbourne 1992).

The Individuals with COPD Exercise Monitoring Schedule designed by the researchers in line with the literature was evaluated in three ways: face to face, telephone and online. The form, which includes seven daily exercises that increase periodically every 2 weeks, includes diaphragmatic breathing and puckered lip exercises taught in the patient education booklet and consists of a follow‐up chart that starts with five breathing exercises in the first week and increases periodically (GOLD 2021, 2023).

### 2.5 Procedure and Measurement

The study was conducted between July 2022 and May 2023. After obtaining the necessary approvals from the ethics committee and the institution, the study was registered in the clinical trial. Ethics committee permission and institutional permission were obtained to conduct the study. The study was registered in ClinicalTrials, and an RCT number was obtained (NCT06128902). Verbal and written consent was obtained from the patients who agreed to participate in the study.

After written and verbal consent was obtained from individuals with COPD who met the inclusion criteria and consented to participate in the study, individuals were assigned to groups. After it was determined which group the individual was in, the pretest was conducted. The pretest data were administered for everyone assigned to the intervention and control groups with the ‘Patient Information Form’ and ‘SF‐36 Quality of Life Scale (Short Form)’ using the face‐to‐face interview technique within an average of 15–20 min. The posttest data of the study were collected by the researchers by telephone after 12 weeks. In the posttest data of the study, the individuals were asked how many times they had been admitted to the hospital due to COPD in the last 12 weeks, and the ‘SF‐36 Quality of Life Scale (Short Form)’ was filled out again.

### 2.6 Intervention

In this study, home‐based follow‐up, counselling and exercise training based on the telecounselling method were applied as interventions.

In the first stage, patients were given exercise training in two sessions, each lasting 20 min. Then, everyone in both the experimental group and the control group received explanation on how to use the follow‐up charts prepared in order to ensure their follow‐up at home. Then, they were told how to use the control cart prepared to facilitate the follow‐up of patients at home.

#### 2.6.1 Intervention Group

Everyone in the intervention group was instructed in exercise training through a two‐session home‐based exercise training programme. The home‐based exercise training content was prepared by the researchers in line with the Turkish Thoracic Society Training Book Series (2019). The training booklet includes training on diaphragmatic and puckered lip breathing and the correct management of the exacerbation period. The training was given to the patients in the intervention group in two sessions averaging 20 min each, one on one in the room where they were hospitalized in the hospital. In the content of the training, techniques such as lecture, discussion, question and answer and demonstration were used. After the training, diaphragmatic and puckered lip breathing exercises were practiced one to one with the patients and continued until the patients performed them correctly. The prepared training booklet was given to the patients in the experimental group after the training.

Home‐based follow‐up was carried out with a control chart prepared by the researchers for the patients in the intervention group. The control chart was designed across 12 weeks separately and each week, day by day. In the tally sheet, patients were asked to mark the exercises they performed daily.

Telecounselling was provided to the patients in the intervention group once a week, 12 times in total, with an average of 10‐ to 15‐min phone calls (Lee and Park 2021; Song et al. 2023). In the telephone interview, the patients' regular exercise status was questioned. The telephone interview was conducted regularly by the researcher every Monday between 17.00 19.00 h. Patients who could not be reached on the day and time of the phone call were called again the next day.

#### 2.6.2 Control Group

The individuals included in the control group of the study were not given any training by the researcher during the study, and these individuals were not followed up by telephone. After the posttest application was completed, the training booklet prepared by the researchers was given to the patients who requested it.

### 2.7 Data Analysis

The evaluation phase of the data was carried out by an expert statistician using the SPSS for Windows 22 package program. In addition to numbers, percentages, minimum and maximum values, mean and standard deviations, Kolmogorov Smirnov test for normal distribution, skewness and kurtosis coefficients, independent groups *t* test, Mann–Whitney *U* test, dependent groups *t* test and Wilcoxon analysis were used in the analysis of the data. The value of *p* < 0.05 was considered statistically significant.

### 2.8 Ethical Considerations

Ethics committee permission and institutional permission were obtained to conduct the study. The study was registered in ClinicalTrials, and the RCT number was obtained (NCT06128902). Written and verbally informed consent was obtained from individuals who agreed to participate in the study. The rules of the Declaration of Helsinki were followed at every stage of the study.

## 3 Results

The patients' sociodemographic and disease characteristics of the individuals are presented in Table 1. Individuals in the experimental and control groups were similar in terms of all control variables (*p* > 0.05) except for age and mean number of COPD exacerbations (*p* < 0.05) (Table 1).

**TABLE 1:** Characteristics of the groups.

	Intervention group (*n* = 40)	Control group (*n* = 40)	Test	*p*
	X ± SD	X ± SD		
Age (year)	64.15 ± 10.72	70.88 ± 11.10	*t* = 2.756	0.007
Number of COPD exacerbations	10.60 ± 5.23	13.75 ± 5.65	*t* = 2.588	0.012
Gender	*n* (%)	*n* (%)		
Female	10 (25.0)	12 (30.0)	*x* ^2^ = 0.251	*p* = 0.617
Male	30 (75.0)	28 (70.0)		
Marital status				
Married	36 (90.0)	38 (95.0)	*p* < 0.001	*p* = 0.675^a^
Single	4 (10.0)	2 (5.0)		
Education level				
Literate	5 (12.5)	12 (30.0)	*x* ^2^ = 4.225	*p* = 0.238
Primary school	32 (80.0)	24 (60.0)		
Middle school	2 (5.0)	3 (7.5)		
High school	1 (2.5)	1 (2.5)		
Employment status				
Working	6 (15.0)	1 (2.5)	*x* ^2^ = 4.291	*p* = 0.117
Not working	10 (25.0)	9 (22.5)		
Retired	24 (60.0)	30 (75.0)		
Income level				
Income less than expenditure	17 (42.5)	18 (45.0)	*x* ^2^ = 3.410	*p* = 0.182
Income equals expenditure	23 (57.5)	19 (47.5)		
Income more than expenditure	—	3 (7.5)		
Insurance				
Yes	34 (85.0)	39 (97.5)	*p* < 0.001	*p* = 0.108^a^
No	6 (15.0)	1 (2.5)		
BKI (kg/m^2^)				
16–20	5 (12.5)	2 (5.0)	*x* ^2^ = 4.684	*p* = 0.096
21–25	30 (75.0)	37 (92.5)		
25 and up	5 (12.5)	1 (2.5)		
COPD Stage				
Gold 1	27 (67.5)	21 (52.5)	*x* ^2^ = 2.917	*p* = 0.233
Gold 2	11 (27.5)	13 (32.5)		
Gold 3	2 (5.0)	6 (15.0)		
Regular checkups				
Yes	39 (97.5)	39 (97.5)	*p* < 0.001	*p* = 1.000^a^
No	1 (2.5)	1 (2.5)		
Medicines used				
Anticholinergic	4 (10.0)	12 (30.0)	*x* ^2^ = 6.654	*p* = 0.155
Beta2antagonist	10 (25.0)	7 (17.5)		
Methylxanthines	—	1 (2.5)		
Corticosteroids	19 (47.5)	13 (32.5)		
Antibiotics	7 (17.5)	7 (17.5)		
Other chronic disease				
Yes	24 (60.0)	26 (65.0)	*x* ^2^ = 0.213	*p* = 0.644
No	16 (40.0)	14(35.0)		
Smoking status				
Never smoked	5 (12.5)	8 (20.0)	*x* ^2^ = 2.876	*p* = 0.237
Smoke	12 (30.0)	6 (15.0)		
Quit smoking	23 (57.5)	26 (65.0)		

Abbreviations: BKI, body mass index; COPD, chronic obstructive pulmonary disease; GOLD, The Global Initiative for Obstructive Lung Disease; *t*, independent two sample test; *x* ^2^, chi‐square test; X ± SD, mean ± standard deviation.

^a^ Fisher's exact test, *p* < 0.05.

The distribution of the number of hospital admissions of the individuals in the experimental and control groups after 12 weeks is presented in Table 2. The difference between the mean number of hospital admissions of the individuals in the experimental and control groups after the intervention (after 12 weeks) was found to be statistically significant (*p* < 0.05).

**TABLE 2:** Distribution of the number of hospital admissions of individuals in the experimental and control groups by group and time after the intervention.

Time	Intervention group		Control group		Test/*p*
	X ± SD	Median (min–max)	X ± SD	Median (min–max)	
Postintervention	1.51 ± 0.77	1 (1–4)	3.26 ± 1.29	3 (1–7)	*t* = 7.190, *p* < 0.001

Abbreviations: *t* independent two sample test, *p* < 0.05; X ± SD, mean ± standard deviation.

Although the mean number of hospital admissions of the experimental group was 1.51 ± 0.77 in 12 weeks, the mean number of admissions of the control group was 3.26 ± 1.29 (Table 2).

The distribution of SF‐36 Quality of Life Scale (Short Form) and subdimension scores of the individuals in the experimental and control groups according to group and time is presented in Table 3. It was determined that there was a statistically significant difference between the mean scores of the physical function, physical role difficulty, emotional role difficulty, energy/vitality/vitality, pain and general health perception subdimensions of the ‘SF‐36 Quality of Life Scale’ of the individuals in the intervention and control groups according to group and time (*p* < 0.05), whereas there was no statistically significant difference in the mean scores of the mental health and social functioning subdimensions (*p* > 0.05) (Table 3).

**TABLE 3:** SF‐36 quality‐of‐life scale and subscale scores of individuals in the experimental and control groups by group and time.

	Time	Intervention group		Control group		Test/*p*
		X ± SD	Median (min–max)	X ± SD	Median (min–max)	
Physical function	Pretest	29.50 ± 26.31	20 (0–90)	24.63 ± 15.75	25 (0–65)	*t* = −1.006, *p* = 0.318
	Posttest	41.71 ± 24.61	32.50 (15–90)	21.03 ± 13.58	20 (0–50)	*t* = −4.550, *p* < 0.001
Test/*p*		*t* = −1.845, *p* = 0.073		t = 0.903, *p* = 0.372		
Physical role difficulty	Pretest	6.25 ± 22.47	0 (0–100)	5.63 ± 19.19	0 (0–100)	U = 784.500, *p* = 0.881
	Posttest	44.08 ± 29.88	50 (0–75)	7.05 ± 17.16	0 (0–75)	*t* = −6.645, *p* < 0.001
Test/*p*		*t* = −6.561, *p* < 0.001		Z = ‐0.988, *p* = 0.323		
Emotional role difficulties	Pretest	5.00 ± 16.10	0 (0–66.67)	3.33 ± 10.13	0 (0–33.33)	U = 783.500, *p* = 0.746
	Posttest	41.23 ± 33.27	33.33 (0–100)	5.98 ± 15.05	0 (0–66.67)	*t* = −5.963, *p* < 0.001
Test/*p*		*Z* = −4.110, *p* < 0.001		*Z* = −1.414, *p* = 0.157		
Energy/vitality	Pretest	39.63 ± 12.32	42.50 (15–60)	34.88 ± 16.51	35 (0–65)	*t* = −1.459, *p* = 0.149
	Posttest	46.97 ± 7.49	47.50 (35–60)	40.26 ± 15.09	45 (5–65)	*t* = −2.484, *p* = 0.016
Test/*p*		*t* = −2.517, *p* = 0.016		*t* = −1.394, *p* = 0.171		
Mental health	Pretest	44.80 ± 11.32	44 (16–64)	43.40 ± 13.39	44 (12–72)	*t* = −0.505, *p* = 0.615
	Posttest	51.47 ± 6.45	52 (32–60)	49.46 ± 13.24	48 (16–92)	*U* = 619.000, *p* = 0.207
Test/*p*		*Z* = −2.862, *p* = 0.004		*Z* = −2.034, *p* = 0.042		
Social functioning	Pretest	36.87 ± 14.41	37.50 (0–62.50)	38.44 ± 16.36	37.50 (0–87.50)	*t* = 0.453, *p* = 0.652
	Posttest	42.43 ± 12.85	50 (12.50–62.50)	41.99 ± 16.84	50 (12.50–75)	*t* = −0.131, *p* = 0.896
Test/*p*		*t* = −1.379, *p* = 0.176		*t* = −1.036, *p* = 0.307		
Pain	Pretest	44.69 ± 21.48	45 (20–100)	44.44 ± 18.39	45 (10–90)	*t* = −0.056, *p* = 0.956
	Posttest	62.96 ± 17.73	67.50(32.50–100)	44.42 ± 17.90	45 (10–90)	*t* = −4.564, *p* < 0.001
Test/*p*		*t* = −4.158, *p* < 0.001		*t* = 0.000, *p* = 1.000		
General health	Pretest	31.63 ± 13.32	32.50 (0–55)	27.50 ± 13.54	27.50 (0–50)	t = −1.374, *p* = 0.174
	Posttest	42.76 ± 5.03	45 (30–50)	32.44 ± 14.00	35 (0–60)	*t* = −4.330, *p* < 0.001
Test/*p*		*t* = −5.442, *p* < 0.001		*t* = −1.745, *p* = 0.089		

Abbreviations: *t*, dependent group *t* test; *U*, Mann Whitney U analysis, *p* < 0.05; X ± SD, mean ± standard deviation; *Z*, Wilcoxon analysis.

## 4 Discussion

The difference between the mean number of hospital admissions of the individuals in the experimental and control groups after the intervention (12 weeks later) was found to be statistically significant (*p* < 0.05). Telerehabilitation practices, which have come on the agenda intensively especially during and after the Covid‐19 period, have also been of great importance for patients with COPD. Tsutsui et al. (2021) emphasized the importance of PR studies applied with the telerehabilitation method in their study in which they explained the new standards related to telerehabilitation in patients with COPD and stated that studies with a high level of evidence should be conducted in this regard. Zanaboni et al. (2017) conducted a study to examine the effect of long‐term telerehabilitation applied to individuals with COPD on repeated hospitalizations and the quality of life of individuals and found that counselling with telerehabilitation was effective in reducing repeated hospital admissions of individuals. In the study conducted by Sahin and Naz (2018) on 212 individuals with COPD who completed an 8‐week exercise programme, it was determined that there was a decrease in the number of hospital admissions and hospitalizations because of the programme. Benzo et al. (2015) reported that PR applied to individuals with COPD after discharge was effective in reducing repeated hospitalizations. Davis et al. (2015), in their study in which they applied PR with telerehabilitation method and compared the hospitalization status of patients with COPD 1, 3 and 6 months after discharge, found that there was a 50% decrease in the hospitalization rate of individuals after 1 month and a 13%–19% decrease in the hospitalization rate after 6 months. Jones et al. (2014) evaluated the hospitalizations of individuals with COPD admitted to the hospital due to acute exacerbations at the end of 12 months following the exercise programme of patients who underwent an exercise programme using telerehabilitation method for 14 days after discharge and found that the number of hospital admissions of individuals decreased. In a study conducted by Cruz et al. (2014) on individuals with COPD, it was determined that home‐based remote monitoring reduced COPD exacerbations and reduced repeated hospitalizations. In the systematic review conducted by Mishra et al. (2024) to examine the impact of telehealth‐based interventions on the length of hospital stay and hospital readmissions of individuals with COPD, a total of 12 randomized controlled trials were analysed. Among these studies, only four were found to reduce hospital readmissions, whereas the others did not provide clear evidence on this matter. This was attributed to the fact that multiple factors could influence hospital readmissions in individuals with COPD. With this finding, which is parallel with the findings of similar studies in literature, it can be considered that telerehabilitation and PR are an important nursing intervention that reduces the repeated hospitalization of patients.

When the mean scores of the subdimensions of the ‘SF‐36 Quality of Life Scale’ of the individuals in the intervention and control groups in the study consisting of individuals with COPD were analysed according to group and time, it was determined that there was a statistically significant difference between the mean scores of the intervention group and the control group in the subdimensions of physical function, physical role difficulty, emotional role difficulty, energy/vitality/vitality, pain and general health perception (*p* < 0.001, *p* < 0.001, *p* < 0.001, *p* = 0.016, *p* < 0.001, *p* < 0.001 and *p* < 0.001, respectively). This difference was related to the fact that the mean posttest scores of the intervention group were higher than the mean posttest scores of the control group. Or et al. (2022) study also mentioned that mobile health applications are developments that can be effectively used, especially in managing the disease conditions of individuals with chronic illnesses. These advancements positively impact patient satisfaction levels. It is natural that an increase in satisfaction leads to an improvement in the quality of life. Zanaboni et al. (2017) conducted a study to examine the impact of long‐term telerehabilitation on the repeated hospitalizations and quality of life of individuals with COPD. They found that counselling provided through telerehabilitation improved the quality of life for individuals with COPD. Hartman et al. (2023) conducted a systematic review of randomized controlled trials on pulmonary rehabilitation administered via telecounselling to individuals with COPD and concluded that telecounselling‐based pulmonary rehabilitation had a positive effect on improving the quality of life of individuals with COPD. Zhang et al. (2022) found that pulmonary rehabilitation training provided through telerehabilitation to individuals with COPD had a positive effect on their quality of life. Cox et al. (2021), in a study involving 1.904 individuals with COPD, determined that exercise training delivered via telephone, video conferencing and video methods significantly improved physical functionality and quality of life. Godtfredsen et al. (2020) identified that pulmonary rehabilitation administered through telecounselling was effective in improving the quality of life of individuals with COPD. Hansen et al. (2020) found that pulmonary rehabilitation delivered via telerehabilitation increased the quality of life of individuals with COPD. Holland et al. (2013) observed an improvement in quality of life in individuals with COPD who were followed up with phone calls after receiving group‐ or home‐based rehabilitation. Tsai et al. (2017) reported that exercise training provided three times a week via telerehabilitation to individuals with COPD enhanced their quality of life. This finding aligns with similar studies in the literature. The improvement in quality of life through pulmonary rehabilitation, considered one of the most critical components of the treatment chain, delivered via telerehabilitation to enable remote access to rehabilitation services, is a finding that supports the literature.

### 4.1 Limitations

Data were collected at the clinic where one of the researchers worked. This constituted the limitation of the study.

## 5 Conclusion

As a result of the study, after the intervention, the number of hospital admissions in the experimental group was significantly lower compared to the control group. Additionally, the SF‐36 Quality of Life Scale revealed significant improvements in the subdimensions of physical functioning, physical role difficulty, emotional role difficulty, energy/vitality, pain and general health perception in favour of the experimental group. However, no significant differences were observed between the groups in the mental health and social functioning subdimensions.

These findings suggest that telecounselling‐based home monitoring, counselling and exercise training are effective in improving quality of life and reducing hospitalizations in individuals with COPD. In line with these findings, it can be recommended that nurses caring for individuals with COPD should perform home‐based exercise practices with telecounselling methods to reduce recurrent hospitalizations and improve the quality of life of these individuals.

## Author Contributions

NK and HD conceptualised the study. NK and HD contributed to the study design and implementation. NK collected the data. NK and HD performed the data analysis. NK and HD wrote the paper with all other authors providing critical review. All authors approved the final version.

## Conflicts of Interest

The authors declare no conflicts of interest.

## Data Availability

Research data are not shared.
